# Large-Scale Production of Isocitric Acid Using *Yarrowia lipolytica* Yeast with Further Down-Stream Purification

**DOI:** 10.3390/biotech12010022

**Published:** 2023-03-13

**Authors:** Svetlana V. Kamzolova, Vladimir A. Samoilenko, Julia N. Lunina, Igor G. Morgunov

**Affiliations:** G.K. Skryabin Institute of Biochemistry and Physiology of Microorganisms, Pushchino Center for Biological Research of the Russian Academy of Sciences, Prospect Nauki 5, Pushchino 142290, Moscow Region, Russia; kamzolova@rambler.ru (S.V.K.); samva@rambler.ru (V.A.S.);

**Keywords:** large-scale biology, yeast *Yarrowia lipolytica*, biosynthesis, isocitric acid (ICA), citric acid (CA), isolation, purification

## Abstract

Isocitric acid (ICA) refers to a group of promising regulators of energy metabolism which has antistress, antihypoxic, and antioxidant activities. In this paper, we reported a process of ICA production from rapeseed oil using yeast *Yarrowia lipolytica* VKM Y-2373 in a 500-L fermentor. The producer synthesized 64.1 g/L ICA with a product yield of 0.72 g/g and a productivity 0.54 g/L·h. We also developed an effective purification method, including a cell separation, clarification, concentration, acidification, and crystallization process, which resulted in the formation of the crystals of monopotassium salt of ICA with a purity of 99.0–99.9%. To the best of our knowledge, this is the first report on an ICA production process at an upscaled bioreactor level.

## 1. Introduction

The isocitric acid molecule has two asymmetric carbon atoms, which leads to the existence of four stereoisomers: *threo*-D_S_-, *threo*-Ls-, *erythro*-Ds-, and *erythro*-Ls- [1]. Among the four isomers, only *threo*-Ds-isocitric acid (ICA) is interesting for organic synthesis and medical applications [2,3]. ICA is recommended for the treatment of iron-deficient anemia, metabolic myopathy caused by defects in cell metabolism, and neoplasms [1,2,3,4,5]. ICA belongs to a group of regulators of energy metabolism and has antistress, antihypoxic, and antioxidant effects [6]. ICA neutralizes the adverse effect of heavy metals on learning and memory, which has been demonstrated in experiments on animals [7,8].

Despite this impressive ICA effect, it is rarely used due to the complexity of its production and high cost. Until now, ICA has been produced using chemical synthesis, resulting in the formation of a mixture of all stereoisomers, which are practically impossible to separate. The naturally occurring stereoisomer ICA from *Sedum spectabile* is sold at a price of USD 279 per 250 milligrams by Sigma–Aldrich.

The microbiological synthesis of ICA using yeast *Yarrowia lipolytica* is more promising as it would allow the production of an exclusive natural *threo*-Ds-stereoisomer. It should be noted that the yeast *Y. lipolytica*, as well as ICA and other products based on its synthesis, are generally recognized as safe (GRAS) and can be used in food, medicine, and pharmacology [9,10].

The literature data describe the processes of ICA production from ethanol [11,12], rapeseed oil [13,14,15], sunflower oil [16,17,18,19], glucose [20], glycerol [20], raw glycerol [21,22,23], and ethanol industry waste [24,25]. The above articles note that *Y. lipolytica* has the ability to synthesize ICA and citric acid (CA) simultaneously in a proportion that depends on the strain, carbon source, and composition of the growth medium. The formation of these acids, rather than other metabolites of Tricarboxylic acid cycle under conditions of nitrogen deficiency, is determined by the high activity of citrate synthase (the enzyme catalyzing the formation of citrate from oxaloacetate and acetyl-CoA) and aconitate hydratase (the enzyme isomerizing citrate to isocitrate) compared to that of subsequent enzymes (nicotinamide adenine dinucleotide (NAD)-dependent isocitrate dehydrogenase, α-ketoglutarate dehydrogenase, and others). The ratio between citric acids toward the ICA production can be shifted via the inhibition of isocitrate lyase (ICL), catalyzing the cleavage of isocitrate to succinate and glyoxylate in the glyoxylate cycle. We reported that the use of the ICL inhibitors such as oxalic and itaconic acids is a simple and convenient method of predominant ICA synthesis from different carbon sources [12,13,14,17,25]. On the contrary, the transformant *Y. lipolytica*, with overexpression of the gene *ICL1*, mainly accumulated CA [18]. Moreover, the level of the dissolved oxygen in the cultivation medium affected the production of CA and ICA, and this dependence was related to the carbon source [26,27,28].

It should be noted that most of the reported studies have been carried out in lab-scale fermenters with volumes ranging from 600 mL to 10 L [11,12,13,14,15,16,17,18,19,20,21,22,23,24,25,26,27,28]; however, there have been no reports of ICA production in pilot-scale bioreactors. There are only a few articles that cover large-scale processes using *Y. lipolytica*, namely, lipase production [29,30]. At the same time, the testing of ICA production technology in large-scale bioreactors is a necessary step towards industrial production. Moreover, the large-scale fermentation process must be established together with the development of an adequate downstream process, ensuring the high purity and yield of the target product. Current electrodialysis-based methods for ICA isolation are quite complex and require multiple steps [2,3]. The improved isolation of ICA has been achieved using adsorption on activated carbon and recovery with methanol [16].

The aim of this work was to develop an ICA production process in a large-scale bioreactor. The isolation of ICA from the culture broth was also investigated to obtain a product of high purity.

## 2. Materials and Methods

### 2.1. Chemicals

All chemicals were of analytical grade (Sigma–Aldrich, St. Louis, MO, USA). Rapeseed oil was purchased from the “Russian seeds” Processing Plant (Vinev, Russia).

### 2.2. Seed Culture

The wild yeast strain *Y. lipolytica* VKM Y-2373 used in this study can produce significant amounts of ICA in media with rapeseed oil [13,14]. The strain was maintained at 4 °C on agar slants with *n*-alkanes as the carbon source.

Cells were first precultivated in a 750-mL Erlenmeyer flask with 100 mL medium with 100 mL of the medium containing (g/L): rapeseed oil, 20; (NH_4_)_2_SO_4_, 3.0; MgSO_4_·7H_2_O, 1.4; Ca(NO_3_)_2_, 0.8; NaCl, 0.5; KH_2_PO_4_, 2.0; K_2_HPO_4_, 0.2; yeast extract “Difco”, 0.5; double Burkholder’s trace element solution at 29 °C for 24 h on a rotary shaker (180–200 rpm). Then, the seed culture (5 mL) was transferred to five flasks containing 100 mL of the above medium and shaken for 48 h; pH was maintained at 4.5–6.0 with an addition of 10% KOH.

### 2.3. Cultivation

The seed culture (500 mL) was inoculated into a 10-L ANKUM-2M fermenter (Institute of Biological Instrumentation of RAS, Pushchino, Moscow region, Russia) with an initial volume of 6 L of the above medium, additionally supplemented with Fe^2+^ ions (1.2 mg/L), thiamine (100 µg/L), baker’s yeast extract (8 mL/L), and itaconic acid (1.3 g/L). Rapeseed oil was added in portions (20 g/L) at the moments when oxygen concentration in the fermenter dropped by 10% (of air saturation) from the basal level; the total amount of oil was 552 g for the entire volume of the cultivation medium in a 10-L fermenter.

For a 500-L fermenter, a second precultivation step was carried out during 24 h in two 10-L fermenters containing 6 L of the above medium. The resulting seed culture (13 L) was inoculated into a 500-L bioreactor SGI-500 (Setric Genie Industriel, Toulouse, France) with a working volume of 220 L so that the initial cellular concentration corresponded to that of the 10-L fermentor. Rapeseed oil was added in the portions by 20 g/L, and the total amount of oil was 23.5 kg for the entire volume of the cultivation medium in a 500-L fermenter.

### 2.4. Specification of 10-L Lab-Scale and 500-L Pilot Scale Fermentors

Lab-scale fermentations were carried out in a 10-L lab-scale ANKUM-2M fermentor (Institute of Biological Instrumentation of RAS, Pushchino, Moscow region, Russia), and the scale-up fermentations were conducted in a 500-L fermentor SGI-500 (Setric Genie Industriel, Toulouse, France).

The value of the volumetric oxygen mass transfer coefficient (*k_L_a*) for most of the aerobic biosynthesis processes is one of the most informative scale up criteria. In preliminary experiments, the value of the oxygen mass transfer coefficient (a) was determined in both fermentor systems using the sulfite method at different working volumes, air flow rates, and stirrer rates. It was shown that conditions with similar oxygen mass transfer rates in ANKUM-2M (maximum *k_L_a* = 490 h^−1^) and SGI-500 (maximum *k_L_a* = 420 h^−1^) fermentors can be achieved under the certain conditions of mixing and aeration. For a 10-L fermentor with a working volume of 6 L, the air flow and stirrer rates were 2 volume of air per volume of culture per minute (vvm) and 1050 rpm, respectively. For a 500-L fermentor with a working volume of 320 L, the air flow rate and the stirrer rates were 1 vvm and 300 rpm, respectively.

The specification of a 10-L fermentor: bottom-driven; type of impeller—turbine of Rushton with impeller diameter of 100 mm; number of impellers—1; number of blades—12; total height—370 mm; tank diameter—220 mm; working volume—6–8.1 L.

The specification of a 500-L fermentor: bottom-driven; type of impeller—turbine of Rushton with impeller diameter of 300 mm; number of impellers—3; number of blades—18; liquid height—1710 mm; tank diameter—650 mm; working volume—220–286 L.

Both fermenter systems were equipped with controllers of pH, the concentration of dissolved oxygen (pO_2_), and temperature (E5CN-Q2MT-500, Omron, Japan).

Both fermenter systems were performed at pH 6.0 using a pH controller (M8832 N, Mostec AQ, Liestal, Switzerland) adjustable by the addition of 20% KOH by peristaltic pump.

The control of pO_2_ was conducted using an oxygen electrode (1050e Mettler Toledo LTD, Urdorkyoto, Switzerland). Before inoculation, the pO_2_ value was adjusted to 100%, using air as the inlet gas at the fermentation temperature and pressure. After inoculation, the air supply was cut off; when the pO_2_ was reduced by less than 50%, the minimum air flow rate was set up. During cultivation, the pO_2_ value of 50–55% (of saturation) was controlled by changing the air flow rate and the stirrer rates. For a 10-L fermentor with an initial volume of 6 L during first hours of cultivation, the air flow and stirrer rates increased from 0.1 to 2 vvm and from 400 to 1050 rpm, respectively. For a 500-L fermentor with an initial volume of 220 L, the air flow rate and the stirrer rates were increased from 0.1 to 1 vvm and from 200 to 300 rpm, respectively.

The temperature (29 °C) was controlled using a regulator (E5CN-Q2MT-500, Omron, Japan). In the pilot fermenter, the temperature was controlled using a temperature controller in an open circuit with solenoid valves on the steam heat exchanger line and the water line.

The cultivation time consisted of 144 h. Daily sampling was carried out to determine the concentrations of biomass, nitrogen, ICA, and CA.

### 2.5. Analyses

Yeast growth was controlled by measuring the dry weight of the biomass, where 1–3 mL of the culture broth was filtered through a membrane filter; yeast cells were washed with n-hexane and then dried under vacuum at 110 °C to obtain a constant weight.

Nitrogen consumption was controlled by measuring the NH_4_^+^ concentration, which was determined potentiometrically using an Ecotest-120 ionometer with an Ekom-NH4 electrode (Econix, Moscow, Russia).

For the analysis of ICA, CA, and other organic acids, the culture broth was centrifuged (8000× *g*, 20 °C, 3 min) for cell removal, and 1 mL of supernatant was diluted with an equal volume of 8% HClO_4_ and centrifuged for protein precipitation. The concentration of acids was measured using high-performance liquid chromatography (HPLC) on an HPLC chromatograph (LKB, Bromma, Sweden) equipped with an Inertsil ODS-3 reversed-phase column (250 × 4 mm, Elsiko, Moscow, Russia) at 210 nm; 20 mM phosphoric acid was used as a mobile phase with a flow rate of 1.0 mL/min; the column temperature was maintained at 35 °C. ICA, CA, and other acids were identified with the corresponding standards (reagents from Sigma–Aldrich (St. Louis, MO, USA).

The concentrations of acids were calculated by calibration with external standards based on peak areas.

### 2.6. Product Isolation and Recovery

Cells were removed from the culture broth on a Sharpless AS-26 B/C flow centrifuge (Alfa Laval, Tumba, Sweden) at a rotational speed of 17,000 rpm. The culture supernatant was clarified through AKS-4 carbon cardboard plates (Strassburger, Westhofen/Rheinhessen, Germany). The native solution was filtered under a vacuum of 60–80 kPa/cm^2^. The clarified supernatant was concentrated in a Simax vacuum evaporator (Praha, Czech Republic) (under vacuum of 80–90 kPa) at a temperature not exceeding 55 °C (hot water heating). At the end of the evaporation procedure, the concentrated solution was served hot into the next stage. Then, the concentrated solution was acidified to pH 3.4–3.5 with 85% (*v/v*) formic acid in distilled water under constant agitation at a temperature below 30 °C.

Then, the crystallization of the acidified concentrated solution was carried out. During crystallization, first, the concentrate was cooled to room temperature during 2–3 h then at a temperature of 0–5 °C for 24–48 h with periodic agitation until the crystals were completely ripened. The separation of crystals was carried out on a Nutch-filter under a vacuum. The washing of the crystals was carried out with distilled water in the volume of 1/3 concentrate, then 50% (*v/v*) hydrolytic technical ethyl in the volume of 1/2 of concentrate, and then 96% (*v/v*) hydrolytic technical ethyl in the volume of 1/8 concentrate. The preparation after crystallization was dried in a vacuum chamber with a constant heated dry air supply, removing moist air to a constant weight. All washing and drying procedures were repeated at the recrystallization step.

The purity of the preparation has been assessed enzymatically using a standard (+)-potassium Ds-*threo*-isocitrate monobasic (Sigma–Aldrich, catalogue number 58790).

### 2.7. Calculations

The calculation of production parameters, such as the specific growth rate (µ), ICA yield from rapeseed oil consumed (Y_P/S_) (g/g), volume productivity (Qp) (g/L·h), and the specific rate of ICA synthesis (qp) (g/g·h) were calculated using the following equations:

(1) $$\mu =\frac{lg\;\left(x_{2}\;-\;x_{1}\right)\;}{t_{2}\;-\;t_{1}},Y_{p}\;=\;p/s,\;Q_{p}\;=\;p/(v\cdot t),\;q_{p}\;=\;p/(x\cdot t),$$

where p is the total amount of ICA (g) in the culture liquid by the end of cultivation; s is the total amount of rapeseed oil consumed (g); x is the total amount of biomass (g) in the culture liquid by the end of cultivation; v is the initial volume of culture liquid (L); t is the cultivation time (h); x_2_ is the concentration of biomass (g/L) at the time t_2_, x_1_ is the concentration of biomass (g/L) at the time t_1_; (t_2_ − t_1_) is the time (h) during which the biomass has increased.

### 2.8. Statistical Analysis

All the data presented represents the mean ± standard deviation of experimental triplicates and two measurements for each experiment using an MS-Excel 2013 Data analysis tool (SE < 12%).

## 3. Results and Discussion

### 3.1. ICA Production in a 500-L Bioreactor

It is well known that the concentration of dissolved oxygen (pO_2_) significantly affects the morphological features, the direction of the synthesis of metabolites, and the biochemical activity of yeast *Y. lipolytica*. When *Y. lipolytica* growth occurred at a low or zero dissolved oxygen concentration, the mycelial and pseudomycelial forms prevailed over the yeast forms, without regard to the nature of the carbon and nitrogen sources used [27,28,31,32,33,34]. An increased value of pO_2_ helps to achieve the predominant synthesis of organic acids (mainly CA) instead of sugar alcohols (mannitol, arabitol, and erythritol) [33]. Usually, the stirrer rates of 200–1000 rpm and air flow rate within the range of 0.24–2.0 vvm generate the pO_2_ values of 30–60% saturation, which favored the synthesis of high-value metabolites in *Y. lipolytica* [26,28,34,35]. We observed that a rise in pO_2_ value from 5 to 60% saturation enhanced ICA production by 3.3 times in *Y. lipolytica* grown on ester-aldehyde fraction in small fermenters [25]. In addition, we found that, in *Y. lipolytica* cultivated at a pO_2_ of 50–55% saturation, the yield of ICA from rapeseed oil (Y_p_) and the specific rate of ICA synthesis (q_p_) were much higher than the values found in the cultures with oxygen deficiency (5–10% saturation) [13].

Based on the above, in the present work, the concentration of pO_2_ was maintained at a saturation level of 50–55%.

For a 500-L bioreactor, this value was provided by varying the air flow rate from 0.1 to 1 vvm and the stirrer rate from 200 to 300 rpm.

**Figure 1:**
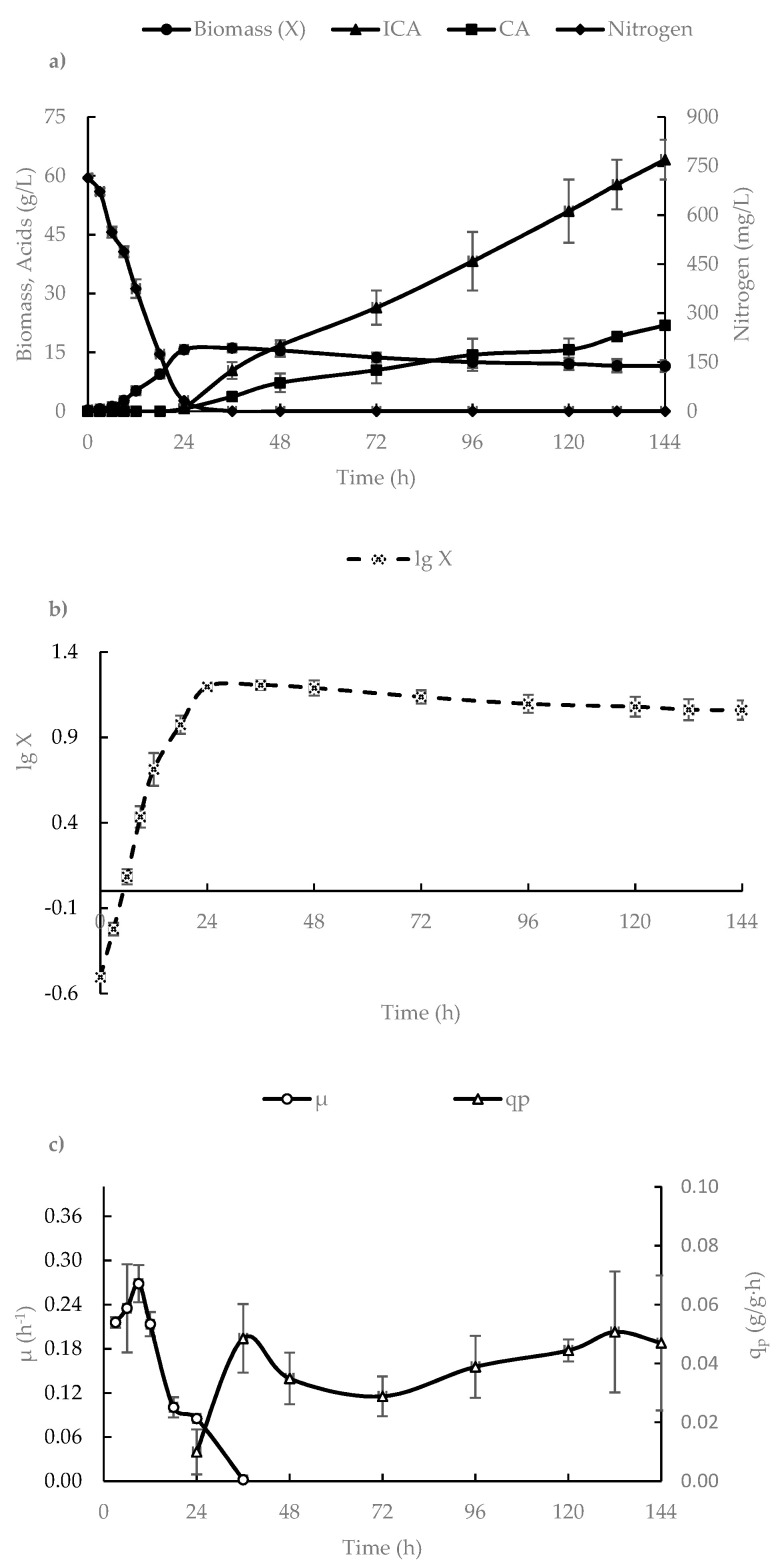
The dynamics of the nitrogen consumption in the medium, biomass (X) accumulation, isocitric acid (ICA), and citric acid (CA) production in *Y. lipolytica* VKM Y-2373 cultivated in a 500-L bioreactor (**a**), the lg X curve (**b**), and the calculated parameters of the specific growth rate (µ) and the specific rate of ICA synthesis (q_p_) (**c**).

Figure 1 presents the dynamics of the growth parameters of *Y. lipolytica* VKM Y-2373, the nitrogen consumption, ICA, and CA production in a 500-L bioreactor.

As seen from Figure 1, until 9 h of cultivation, the growth of *Y. lipolytica* VKM Y-2373 was linear (exponential phase), since nutrients are in plentiful supply; the value of the maximum specific growth rate (µ_max_) was 0.27 h^−1^. Starting at 9 h of cultivation, the cells passed into a phase of retarded growth caused by the depletion of nitrogen from the medium; the value of µ was gradually decreased, and, by 24 h, the culture passed into the stationary phase, when cell growth had completely stopped. The final biomass reached 11.5 g/L. In the exponential and retardation growth phases, there was no excretion of ICA into the medium; ICA excretion began in the stationary phase.

As shown in Figure 1, the specific rate of ICA synthesis (qp) was maintained almost unchanged (0.03–0.05 g/g·h) during the whole stationary phase. At 144 h, *Y. lipolytica* VKM Y-2373 synthesized 64.1 g/L ICA and 21.9 g/L CA. It should be noted that the specific rate of ICA synthesis (qp) began to decrease after 144 h of cultivation (data not shown). This can be due to the inhibitory action of ICA or the aging of cells suffering from the deficiency of nitrogen in the medium. This fact shows that the optimal duration of cultivation is 144 h.

For comparison, *Y. lipolytica* VKM Y-2373 was cultivated in a 10-L fermenter with an initial volume of 6 L. Aeration was maintained at the same level (50–55% saturation) by varying the air flow rate from 0.1 to 2 v/v/min and the agitation rate from 400 to 1050 rpm.

**Figure 2:**
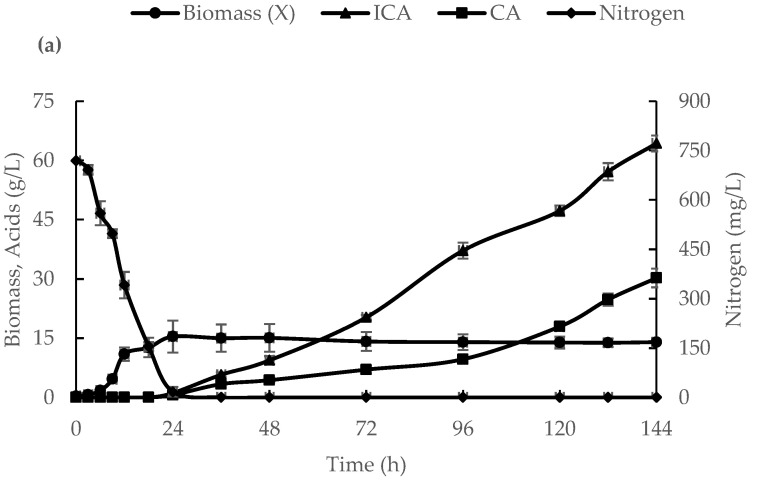

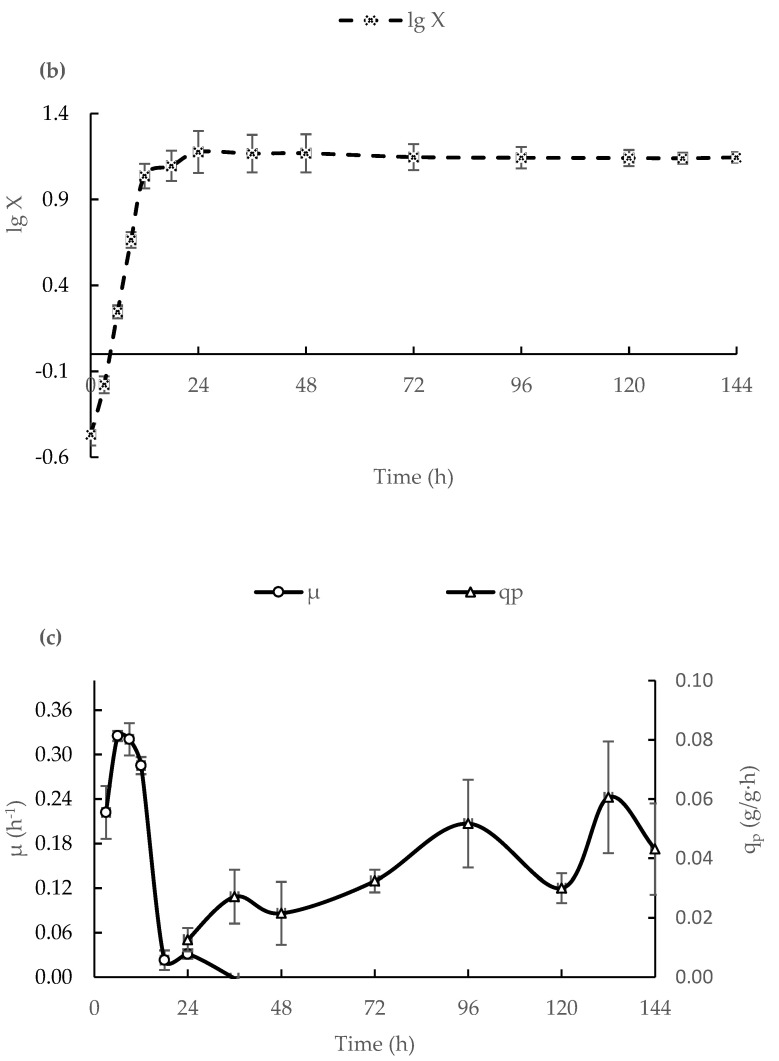
The dynamics of the nitrogen consumption in the medium, biomass (X) accumulation, isocitric acid (ICA), and citric acid (CA) production in *Y. lipolytica* VKM Y-2373 cultivated in a 10-L fermenter (**a**), the lg X curve (**b**), and the calculated parameters of the specific growth rate (µ) and the specific rate of ICA synthesis (qp) (**c**).

As shown in Figure 2, a similar growth pattern was observed under these conditions, with a maximum specific growth rate (µ_max_) of 0.31 h^−1^. The biomass concentration was increased to 14 g/L (22% compared to a 500-L bioreactor). After 24 h of *Y. lipolytica* VKM, Y-2373 actively synthesized acids; moreover, at 144 h, the culture produced the same amount of ICA (64.3 g/L), while the concentration of CA increased to 30.2 g/L (38% compared to a 500-L bioreactor). The reason for the difference in the ICA/CA ratio in small and large fermenters under the same growing conditions and medium composition is not yet understood. Subsequent studies of the processes of the synthesis and secretion of ICA at the biochemical level could shed some light on the reasons for this phenomenon.

Table 1 shows the comparative data on the amounts of synthesized products, the consumed oil, the ratio between ICA and CA, as well as the values of the ICA yield from rapeseed oil consumed (Y_P/S_), q_p_, and the volume productivity (Q_p_) in 500- and 10-L bioreactors. As seen from Table 1, in a 500-L bioreactor, the ratio of citric acids was shifted towards the formation of ICA to 2.9:1, compared with a ratio of 2.1:1 on a 10-L fermenter. The values of Q_p_ and q_p_ did not depend on the volume of the bioreactor and amounted to 0.54–0.6 g/L·h and 0.03–0.04 g/g·h, respectively. However, upscaling the ICA production process decreased the yield from 0.94 g/g of a 10-L culture to 0.74 g/g for a 500-L culture.

It should be noted that the production parameters presented in Table 1 corresponded to the literature data for other *Y. lipolytica* strains, cultivated in a lab-scale fermentor. Thus, the recombinant strain *Y. lipolytica* with a high-level expression of the *ACO1* gene coding aconitate hydratase, which processes the shift of the CA/ICA product pattern into the direction of ICA [19], produced 68.4 g/L ICA and 22 g/L CA with a volume productivity (Q_p_) of 0.46 g/L·h in a 20-L bioreactor with a working volume of 15 L at the dissolved oxygen concentration of 60% saturation [16]. However, this strain increased the ICA/CA ratio by only 11–15% on glucose, sucrose, or glycerol [19]. Other recombinant strains with deleted isocitrate lyase increased ICA/CA ratio by only 2–5% in comparison with the wild strain [18]. The most efficient process of ICA production was realized when using the strain *Y. lipolytica* YALI0E34672g with the enhanced expression of the gene coding for the mitochondrial dicarboxylate–tricarboxylate carrier. This strain provided an accumulation of 136.7 g/L ICA with a process selectivity of 88.1% [20].

### 3.2. Isolation and Purification

Isocitric acid (ICA) was isolated from the monopotassium salt of ICA (K-ICA) directly from the filtrate of the culture broth without preliminary separation of the free acid. Table 2 indicates the characteristics of the yields and purity of K-ICA of different samples during the purification procedure.

As seen from Table 2, at the first stage, the cells were separated from the culture broth. Next, the culture supernatant was clarified and filtrated. After separation and clarification, the volume of the supernatant was decreased due to the removal of biomass and mechanical losses of the culture broth.

Next, the supernatant was concentrated up to 290–390 g/L ICA. As seen from Table 2, the high yields (67–71.4%) of the purified product were obtained within a range of 340–390 g/L ICA. It should be noted that the ultra-deep evaporation contributes to the partial crystallization of the product in an evaporator, which further complicates the unloading of the concentrate and leads to the loss of part of the product. If the concentrate is more dilute, then more ethanol will be required at the product crystallization stage.

Then, the concentrated solution was acidified to pH 3.4–3.5 with 85% formic acid. It should be noted that it is more convenient for large volumes of culture liquid to be evaporated to the maximum concentration before acidification.

Because the most complete recovery of K-ICA from solutions requires high saturation (concentration), this contributes to the contamination of the product with impurities (deposition) and its loss. For this reason, it is better to crystallize K-ICA from water–ethanol mixture, as described in “Materials and Methods”.

After crystallization, a product of technical-grade quality with yields of 58.6–73.2% from the initial concentration of ICA in the cultural broth, and with a base substance content of 90.9–92.0%, was obtained (Table 2).

To increase the purity of K-ICA, a technical salt was recrystallized from a water–ethanol mixture. Salt losses at recrystallization were low (5–12%) when using concentrates of 350–390 g/L ICA and high (22.5%) with a concentrate of 290 g/L ICA. The yield of the purified product after recrystallization was high (67–71.4% of original) when using concentrates of 350–390 g/L ICA and low (49% of original) with a concentrate of 290 g/L ICA. The base substance content of all recrystallized K-ICA preparation consisted of 99.0–99.9%.

It should be noted that there is little information in the literature about the methods of ICA purification. Until now, chemical synthesis has been used to create ICA, which results in four different stereoisomers that are very difficult to separate. Only one of them, threo-Ds-isocitric, is a natural biologically active compound. This stereoisomer is of practical interest since the other three stereoisomers are not metabolized by cells and even inhibit some enzyme systems. Treo-Ds-ICA accumulates in large amounts in plants, fruits, and berries, especially blackberries. Sigma–Aldrich (St. Louis, MO, USA) produces the monopotassium salt of ICA (CAS No. 20226-99-7) from plant material, and the purity of the reagent is more than 98%; however, the price of this reagent is high (889 USD per gram).

It is reported that ICA can be isolated as a trimethyl ester of isocitrate from culture liquid containing almost equivalent amounts of ICA and CA [2,3]. The isolation procedure included the electrodialysis to remove cations such as Na^+^ from the fermentation solution with subsequent acidification of dialysate and the evaporation of liquid at high pressure. Then, the resulting product was treated with an excess of methanol, and the insoluble CA trimethyl ester precipitated as crystals; however, soluble and practically pure ICA trimethyl ester with yields of 80–88% was subjected to a series of further conversions after the formation of lactones and other promising chiral derivatives. Another method was connected with the selective adsorption of ICA and CA directly from the fermentation solution on activated carbon, followed by the isolation of both acids using elution with methanol and their final separation through known methods [16]. This approach avoids the expensive electrodialysis and evaporation and saves time and energy.

## 4. Conclusions

This research was a first attempt to produce isocitric acid (ICA) in a large-scale bioreactor. To date, no such studies have been published in the literature. The producer *Y. lipolytica* VKM Y-2373 synthesized 64.1 g/L ICA from rapeseed oil with a product yield (Y_p_) of 0.72 g/g and volume productivity (Q_p_) of 0.54 g/L·h in a 500-L bioreactor. The successful implementation of an ICA production process in a 500-L bioreactor is due to the maintenance of the same composition of the nutrient medium and cultivation conditions as in a 10-L fermenter. ICA, such as potassium salt, was isolated from the culture broth filtrate in a crystalline form. The subsequent isolation procedures involved the separation, clarification, filtration, concentration, acidification, and ICA extraction with a water–ethanol mixture from the residue. The crystalline monopotassium salt of ICA was characterized by a high degree of purity (99.0–99.9%) and can be used for organic synthesis and medicine.

## Figures and Tables

**Table 1:** Calculated ICA production indices of *Y. lipolytica* VKM Y-2373.

Indices	Volume of Bioreactor	
	500 L	10 L
Initial volume of cultural broth (L)	220.00 ± 0.00	6.00 ± 0.00
Final volume of cultural broth (L)	266.70 ± 21.20	8.05 ± 0.07
Total amount of biomass (g)	3096.00 ± 639.73	112.35 ± 6.65
Total amount of ICA (g)	17,164.20 ± 2687.33	516.46 ± 22.66
Total amount of CA (g)	5847.87 ± 609.84	242.92 ± 21.84
ICA/CA ratio	2.9:1	2.1:1
Rapeseed oil consumed (g)	23,245.33 ± 2329.86	552.00 ± 0.00
Y_p_ (g/g)	0.74 ± 0.05	0.94 ± 0.04
q_p_ (g/g·h)	0.04 ± 0.00	0.03 ± 0.00
Q_p_ (g/L·h)	0.54 ± 0.08	0.60 ± 0.03

Y_p_—ICA yield from rapeseed oil consumed, Q_p_—the volume productivity; q_p_—the specific rate of ICA synthesis.

**Table 2:** Characteristics of yield and purity of monopotassium salt of ICA.

Stages	Indicators	Samples					
		1 *	2 *	3 *	4 **	5 **	6 **
**Fermentation**	Volume of cultural broth (L)	286	244	270	7.9	8.1	8.1
	ICA (g/L)	69.1	59.0	64.2	66.3	62.3	64.3
	CA (g/L)	22.4	21.30	22.0	32.7	28.0	30.0
	Calculated amount of K-ICA (kg)	23.68	17.25	20.77	0.620	0.605	0.620
**Separation** **Clarification** **Filtration**	Volume of supernatant (L)	274	232	258	7.8	7.9	7.8
**Concentration**	Volume of concentrate (L)	54	35	47	1.8	1.5	1.47
	ICA (g/L)	350	390	350	290	340	340
**Acidification**	pH of concentrate after acidification	3.4	3.5	3.4	3.4	3.5	3.5
**Crystallization** **Washing** **Drying**	K-ICA content in technical-grade product (%)	92.0	90.9	92.0	92.0	92.0	92.0
	Amount of technical-grade K-ICA (kg)	18.85	13.3	16.4	0.40	0.48	0.49
	Yield of technical-grade salt (% of the original)	73.2	69.4	72.6	58.6	73.0	72.2
**Recrystallization**	Purity of product (%)	99.9	99.9	99.0	99.0	99.0	99.0
	Amount of K-ICA (kg)	17.07	11.73	15.0	0.31	0.43	0.45
	Yield of purified product (% of the original)	71.0	67.0	71.0	49	71.3	71.4

K-ICA—monopotassium salt of isocitric acid; *—the experiment was performed using a 10-L fermentor; **—the experiment was performed using a 500-L fermentor.

## Data Availability

Data used to support the findings of this study are available from the corresponding author upon reasonable request.
